# Response to comments on ‘Milk ladder versus early oral immunotherapy in infants with cow's milk protein allergy’

**DOI:** 10.1002/clt2.70001

**Published:** 2024-10-22

**Authors:** Chisato Inuo, Yurika Matsumoto

**Affiliations:** ^1^ Department of Allergy Kanagawa Children's Medical Center Yokohama Kanagawa Japan

Dear Editor,

We would like to thank the authors for their thoughtful and constructive comments on our publication, “Milk ladder versus early oral immunotherapy in infants with cow's milk protein allergy.” We appreciate their interest and engagement in this clinical discussion on the management of cow milk protein allergy (CMPA).

The use of dietary advancement therapies (DATs) such as milk and egg ladders is becoming widespread in the global allergy community. It is important to collect and share local data on these practices to expand our collective knowledge of the treatment of food allergies.

As the authors rightly pointed out, our study was conducted in a real‐world clinical setting where, due to various practical constraints, we used parent‐reported clinical histories and sensitization markers instead of the gold‐standard Oral Food Challenge (OFC) for most patients. Data on patients who only had previous immediate allergic reactions due to the ingestion of cow milk protein is shown Table S1. The remaining patients had high levels of cow milk‐specific IgE (>5 kU_A_/L), which exceeded 95% of the predicted value for diagnosing CMPA. We acknowledge that this is a limitation and agree that the absence of a challenge‐proven allergy could have led to the inclusion of some children with asymptomatic sensitization or those with parent‐reported symptoms without OFC. Nonetheless, we aimed to reflect the reality of clinical practice in which OFC is not always feasible or conducted in every case.

We also concur with the authors' insightful differentiation between the primary, secondary, and tertiary allergy prevention and the need to carefully classify patients undergoing milk introduction based on their clinical history and sensitization status. The suggestion that some patients in our study may have undergone the primary or secondary prevention is an important consideration. This underscores the complexity of managing food allergies in clinical settings where treatment protocols often overlap.

In agreement with the authors, we recognize that tolerance and reintroduction outcomes must be carefully labeled, particularly in the absence of prior confirmation of allergy through the OFC. We believe that further studies employing more rigorous diagnostic criteria will be crucial to refine treatment protocols and ensure that true allergic responses are addressed in our interventions.

Once again, we appreciate the authors' valuable feedback and our opportunity to further clarify and discuss the important aspects of CMPA management. We hope that our study contributes meaningfully to the ongoing advancement of DATs, such as Milk Ladder and Early Oral Immunotherapy, and we welcome further comments and discussions in this field.

## AUTHOR CONTRIBUTIONS

**Chisato Inuo**: Writing—original draft. **Yurika Matsumoto**: Writing—review and editing.

## Supporting information

Table S1

