# Supplementary material for: Response to comments on ‘Milk ladder versus early oral immunotherapy in infants with cow's milk protein allergy’
Source: Clin Transl Allergy. 2024 Oct 22;14(10):e70001. doi: 10.1002/clt2.70001 (PMC11496043; doi:10.1002/clt2.70001)
Supplement: Supplementary file 1 — Table S1 [file CLT2-14-e70001-s001.docx]

**Table S1**. Baseline patient characteristics

|  | Milk ladder | Early Oral immunotherapy | *P* value |
| --- | --- | --- | --- |
| Background |  | | |
| Number of patients | 38 | 51 |  |
| History of anaphylaxis (%) | 3 (8) | 0 | 0.07 |
| History of milk allergy (%) | 33(87) | 47(92) | >0.99 |
| Cow’s milk sIgE (kU_A_/L), median (IQR) | 19.35 (0.91–89.5) | 8.04 (0.13–73.3) | 0.008 |
| Casein sIgE (kU_A_/L), median (IQR) | 17.90 (0.22–100) | 6.89 (0.08–100) | 0.07 |

Abbreviation: sIgE, specific IgE
